# Intramedullary nail prior to flap coverage may not increase complications in Gustilo–Anderson Grade IIIB and IIIC open tibial fractures: A retrospective study

**DOI:** 10.1002/jeo2.70522

**Published:** 2025-11-14

**Authors:** Emily Morris, Jarrod Younger, Matthew Hope, Richard Steer, Ahmed Mahmoud

**Affiliations:** ^1^ School of Medicine and Dentistry Griffith University Gold Coast Queensland Australia; ^2^ Department of Orthopaedic Surgery Gold Coast University Hospital Southport Queensland Australia; ^3^ Faculty of Health, Medicine and Behavioural Sciences The University of Queensland Herston Queensland Australia

**Keywords:** intramedullary nail, open fracture, orthopaedic surgery, tibia, trauma

## Abstract

**Purpose:**

This study aimed to assess whether immediate intramedullary nailing and delayed flap coverage increases complications of severe open tibial fractures. Current standards for care indicate temporary external fixation, followed by definitive stabilisation at the time of flap coverage within 7 days of injury. However, this approach can be difficult to coordinate for resource‐constrained centres. Earlier intramedullary nailing and allowing for delayed flap coverage may lead to easier coordination of care.

**Methods:**

Patients were recruited from a trauma database between 2015 and 2024. Those included were over 18 years old with a Gustilo–Anderson IIIB or IIIC open tibia fracture and at least 6 months follow‐up. Patients were grouped by those who had an intramedullary nail within 24 h with delayed soft tissue coverage (Group 1) and those who received initial external fixation and an intramedullary nail at the time of flap (Group 2). Collected complications were deep infection, aseptic nonunion, flap failure and revision surgery. This study equates to level III evidence.

**Results:**

Group A (*n* = 34) and Group B (*n* = 115) were analysed. Gender, smoking status, age and injury severity score were not significantly different. No significant difference was found in outcomes of deep infection rate *χ* ^2^(1, *N* = 149) = 0.08, *p* = 0.78, nonunion *χ* ^2^(1, *N* = 149) = 0.43, *p* = 0.51, flap failure *χ* ^2^(1, *N* = 149) = 0.35, *p* = 0.56 or revision surgery *χ* ^2^(1, *N* = 149) = 1.7, *p* = 0.19. A binary logistic regression showed no difference in infection rates when controlling for confounders (odds ratio [OR] = 1.14, 95% confidence interval [CI]: 0.46–2.85, *p* = 0.780).

**Conclusion:**

Intramedullary nailing within 24 h, without simultaneous flap coverage, does not appear to lead to increased complications in Gustilo–Anderson Type IIIB/C open tibia fractures. However, prospective studies with larger sample sizes are required to confirm these findings.

**Level of Evidence:**

Level III.

**Abbreviations**
AO/OTA: Arbeitsgemeinschaft für Osteosynthesefragen/Orthopaedic Trauma Association
CT: computed tomography
GA: Gustilo–Anderson
IM: intramedullary
IMN: intramedullary nail
IQR: interquartile range
ISS: Injury Severity Score
MBA: motorbike accident
MVA: motor vehicle accident
SD: standard deviation
SPSS: Statistical Package for the Social Sciences
STROBE: Strengthening the Reporting of Observational Studies in Epidemiology

## INTRODUCTION

Gustilo–Anderson (GA) type IIIB and IIIC open tibial fractures are a challenging subset of orthopaedic injuries involving severe soft tissue damage and bone exposure, requiring soft‐tissue flap coverage [6]. A coordinated ortho‐plastic approach is required, with early fixation and closure having optimal outcomes [5, 9, 16].

A key issue when managing these fractures is the timing of intramedullary nail (IMN) placement after initial debridement [7, 8, 12]. The Arbeitsgemeinschaft für Osteosynthesefragen (AO) Foundation recommends the use of initial external fixation to allow the observation of soft‐tissue trauma prior to committing to IMN [1]. This recommendation stems from early research that highlighted concerns regarding increased infection risk and potential disruption of the local blood supply during the fixation process [16]. Another study [13] describes the concept of the ‘second hit’ in trauma management, which contends that immediate surgical intervention may amplify the systemic proinflammatory response in patients with multiple injuries, potentially exacerbating post‐traumatic complications; however, this study proposes modern trauma management requires daily physiology assessment and decisions to be made on a patient‐by‐patient basis.

In contrast, immediate nailing has been advocated by some studies for the potential benefits of preserving the vascularity of soft tissues, decreasing the likelihood of infection, minimising invasive interventions and minimising the time to stabilization [7, 11, 12]. One study demonstrates favourable outcomes through an early ‘fix‐and‐flap’ approach within 72 h that avoids temporary external fixation [11]. However, this article still recommends a coordinated ortho‐plastic procedure over a decoupled one. Execution of this strategy remains inconsistent and is largely hindered by patient factors, resource constraints such as limited access to microsurgical expertise, scheduling of operating theatres and the logistics of inter‐department collaboration [4, 14, 19]. These barriers undermine the intended benefits, leading to higher complication rates when the recommended 72‐h window for soft tissue reconstruction is not met [19].

Open tibial fractures demand time‐sensitive management. Several studies demonstrate that early flap coverage significantly improves postoperative outcomes [8, 9]. However, there remains scope to explore whether de‐coupling ortho‐plastic involvement, through immediate IMN and allowing delayed flap coverage, impacts outcomes in these injuries. Therefore, this study aims to challenge the existing practice of the simultaneous ‘fix‐and‐flap’ approach to determine whether immediate nailing with delayed flap coverage can serve as an acceptable alternative for the management of severe open tibial fractures. This would provide a much‐needed reflection on the current management theory and potentially increase treatment efficiency and expedite patient recovery. The hypothesis of this study is that immediate IMN followed by delayed flap coverage does not lead to significantly higher rates of infection compared with standard management, while the null hypothesis posits that immediate IMN with delayed flap coverage will lead to significantly higher rates of infection compared with standard management.

## METHODS

**Figure 1:**
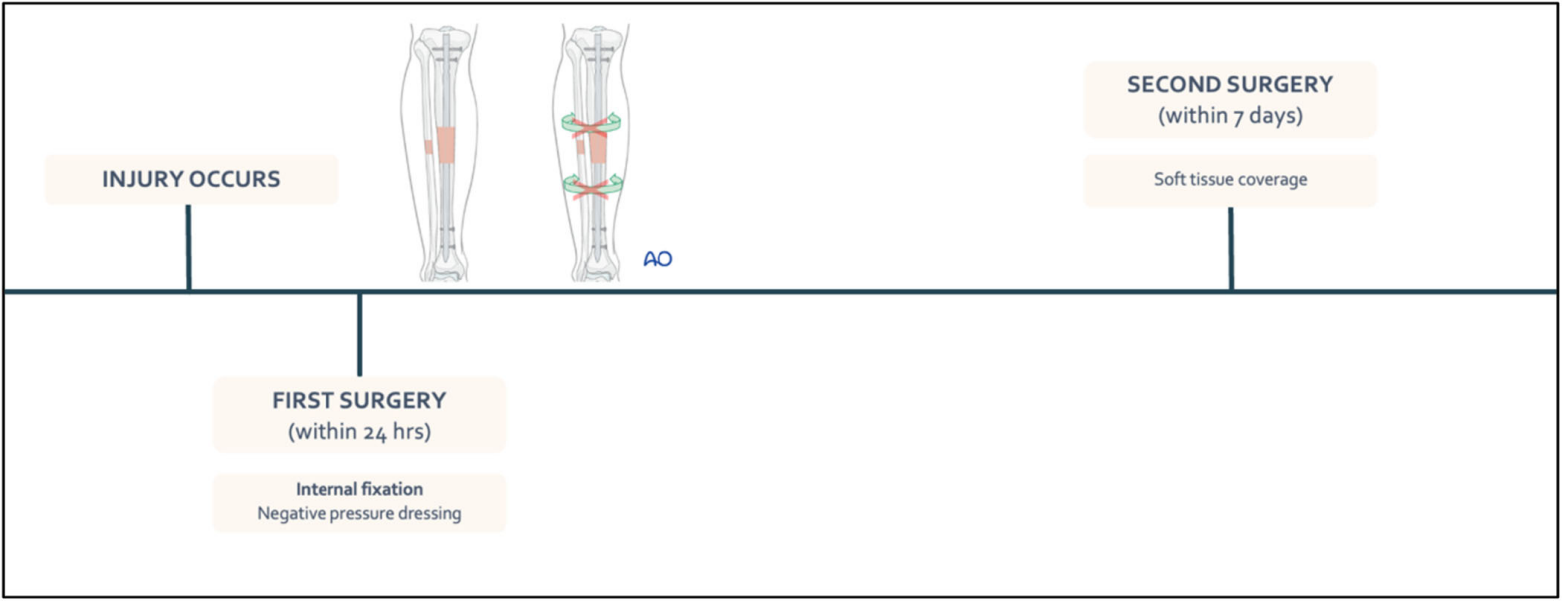
Timeline of immediate tibial nailing (Group 1) [2].

**Figure 2:**
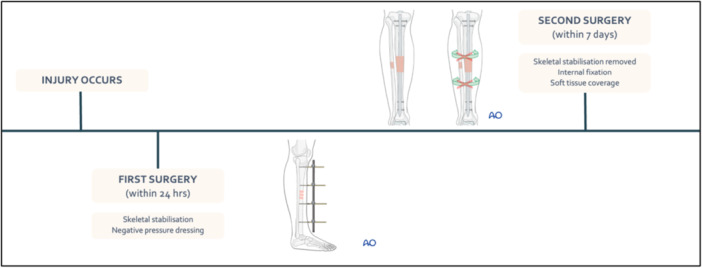
Timeline of delayed tibial nailing (Group 2) [2, 3].

Ethical approval to review the patient's charts for long‐term follow‐up was gained from the Hospital Human Research Ethics board (Approval number LNR/2019/QMS/58750). A retrospective cohort study, representing level III evidence, was conducted to compare the outcomes of two groups of patients: Group 1, who received a two‐stage approach consisting of immediate IMN within 24 h of injury followed by delayed soft‐tissue coverage (see Figure 1); and Group 2, who underwent temporary external fixation, followed by a single‐stage ‘fix‐and‐flap’ approach with IMN and soft‐tissue coverage (see Figure 2).

Data were gathered from a consecutive series of patients admitted to a tertiary trauma centre in Australia with open tibia fractures who were recruited from a prospectively collected trauma database from 2015 to 2024. Patients included were over 18 years of age with an open tibial fracture requiring soft tissue coverage (GA grade IIIB or IIIC) and a minimum follow‐up of 6 months. Excluded were patients with incomplete data or bilateral open tibial fractures. Potential confounders such as age, sex, smoking status, Injury Severity Score (ISS), Flap type, Arbeitsgemeinschaft für Osteosynthesefragen/Orthopaedic Trauma Association (AO/OTA) classification and days to soft tissue coverage were collected. The Strengthening the Reporting of Observational Studies in Epidemiology (STROBE) reporting checklist for observational studies was used for this study. A completed checklist is provided in Supporting Information S1.

The two treatment approaches observed in this cohort reflect pragmatic adaptations to clinical and institutional constraints. Immediate IMN with delayed flap coverage was performed when definitive fixation was prioritised, and soft tissue reconstruction was delayed due to limited plastic surgery availability or theatre access. Initial external fixation followed by combined IMN and flap coverage (‘fix‐and‐flap’) was used in a majority of cases along treatment guidelines. The patients' records, imaging and follow‐up documentation were reviewed to collect the demographics data and outcomes.

The clinical outcomes of interest were deep infection, aseptic nonunion, flap failure, revision surgery and the need for amputation. Aseptic nonunion was defined as the absence of bridging callus in at least three cortices on radiographs or computed tomography (CT) imaging at 6 months postadmission. Deep infection was defined as infection involving bone, confirmed by positive tissue cultures. Flap failure was defined as partial or complete necrosis or the need for return to theatre for flap revision. Amputations (below‐ or above‐knee) were undertaken in cases of persistent infection despite treatment or in the presence of a painful, nonfunctional limb.

**Figure 3:**
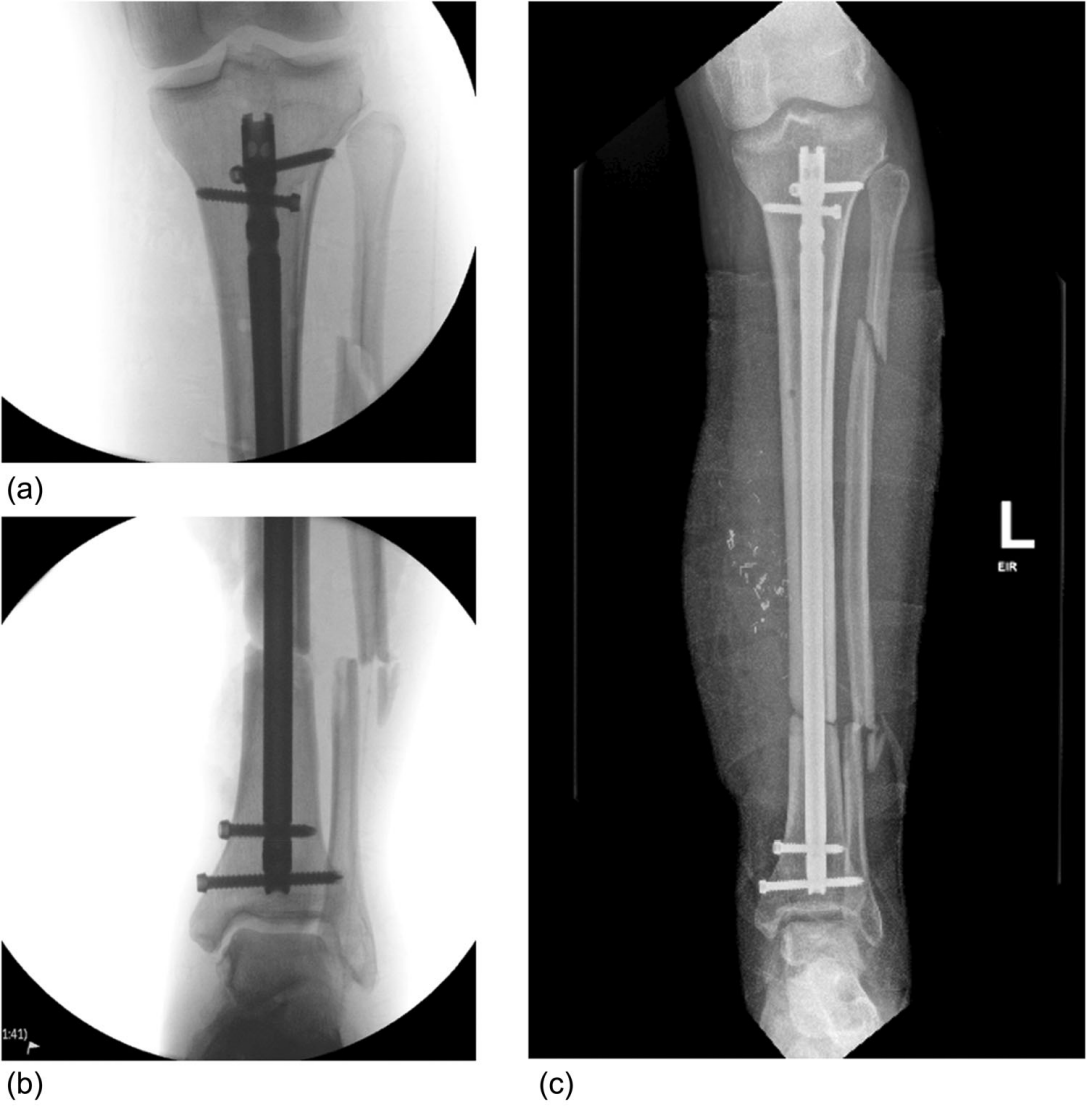
Forty‐seven‐year‐old male sustained a distal tibial diaphyseal fracture from a concrete block. (a, b) Intraoperative anterior posterior (AP) X‐ray images of definitive internal fixation via intramedullary (IM) nail. (c) AP X‐ray with a complete view of the same IM nail.

All patients were managed according to defined standards. A 2 g dose of intravenous cefazolin was administered within 1 h of emergency department presentation and continued for 24 h. Tetanus prophylaxis was provided to patients with uncertain immunisation status. In cases of severe soft tissue loss, oral antibiotics were continued for 5 days or until soft tissue coverage was achieved. Surgical debridement was performed within 24 h of presentation. In Group 1, following debridement, patients underwent definitive fixation with a statically locked intramedullary tibial nail (antegrade, suprapatellar or infrapatellar approach selected by fracture location), such as in Figure 3.

**Figure 4:**
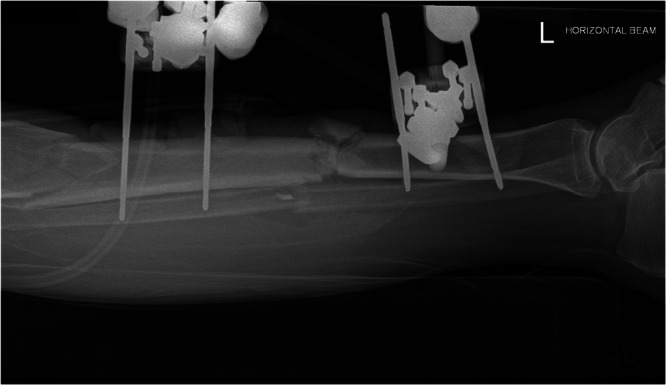
Lateral X‐ray of modular external fixation in situ in a 62‐year‐old male who suffered a comminuted diaphyseal tibial fracture postmotor vehicle accident.

Reaming was used selectively, depending on contamination and soft‐tissue envelope. Blocking screws were employed in proximal/distal fractures to prevent malalignment. Definitive flap coverage (local or free) was then performed by plastics at the earliest safe opportunity (aiming for ≤72 h). In Group 2, a temporary modular external fixation was applied after debridement, like that shown in Figure 4. Pins were inserted in safe corridors away from anticipated flap zones. These frames allowed wound access and limb nursing.

**Figure 5:**
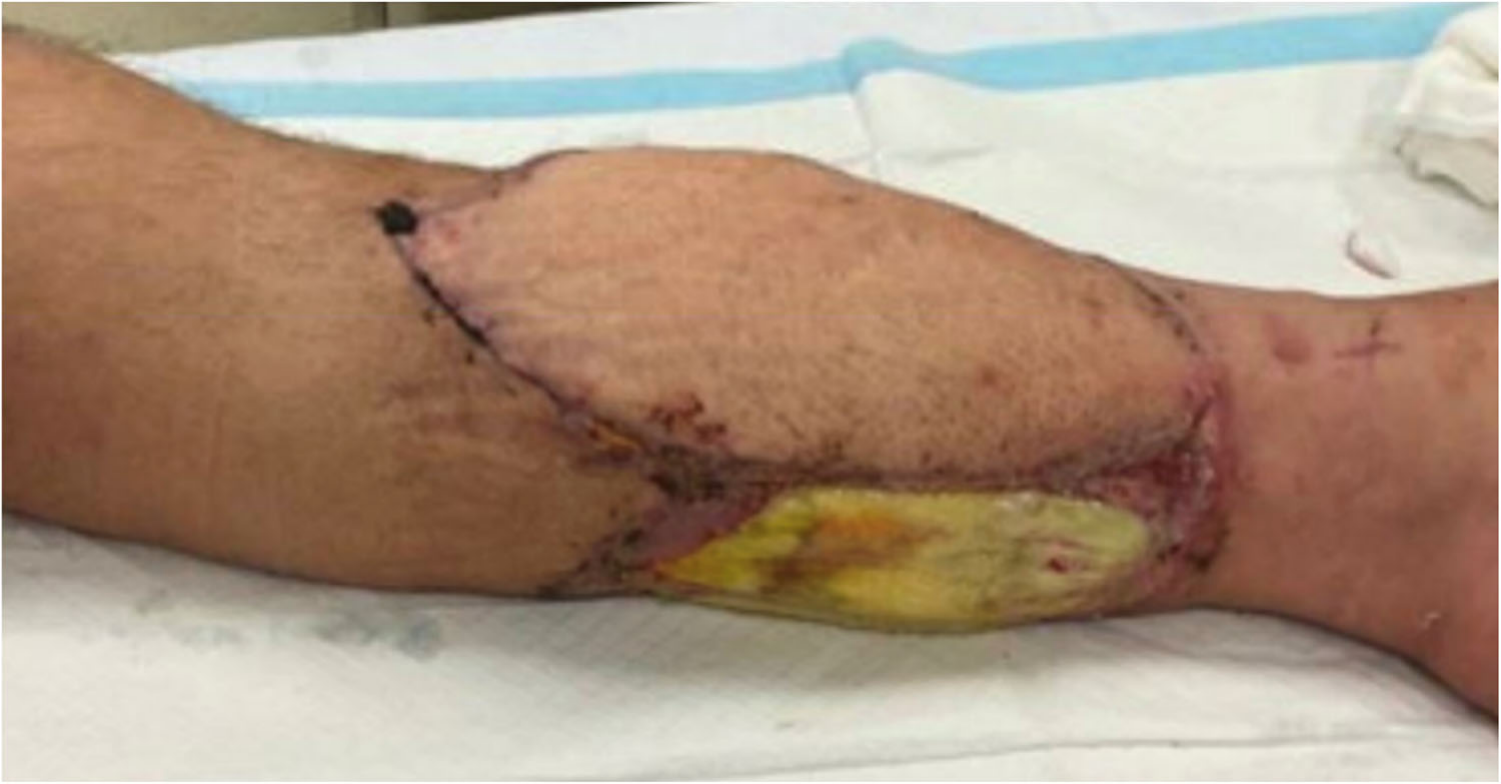
Definitive coverage of an anterolateral thigh free flap in place on an open tibial fracture of a 47‐year‐old male sustained a distal tibial diaphyseal fracture from a concrete block.

At the planned definitive stage, external fixator and pin tracts were removed, and intramedullary nailing was performed, as shown in Figure 4 (with the same technical principles as Group 1). In the same sitting, definitive flap coverage (local rotational or free flap) was carried out by plastics, an example of which is shown in Figure 5.

For both groups, negative pressure wound therapy was applied following initial debridement until definitive coverage and fixation, and maintained until soft tissue coverage had occurred. Antibiotics were continued for 72 h after soft tissue coverage.

### Statistical analysis

Statistical analysis was performed using SPSS Statistics v30 (SPSS Inc.). The primary outcome was the rate of deep infection, while secondary outcomes included revision surgery, aseptic nonunion and flap failure. Differences in outcomes between Groups 1 and 2 were assessed using the Pearson *χ* ^2^ test. Demographic data were analysed using the Mann–Whitney *U* test for non‐normally distributed continuous variables, the *χ* ^2^ test for categorical variables and the Fisher's exact test for categorical variables with a predicted count less than five in more than 20% of cells. Binary logistic regression was conducted to evaluate the effect of potential confounding factors on the deep infection rate. Statistical significance was set at *p* < 0.05. To avoid overfitting in multivariable analyses due to the small number of events, the number of covariates in each logistic regression model was limited to three, in accordance with recommended events per variable (EPV) thresholds (EPV > 10). Two separate models were created, alternately excluding age or smoking while including the other three prespecified confounders (fracture location, flap type and the infection rate).

## RESULTS

### Demographics

**Figure 6:**
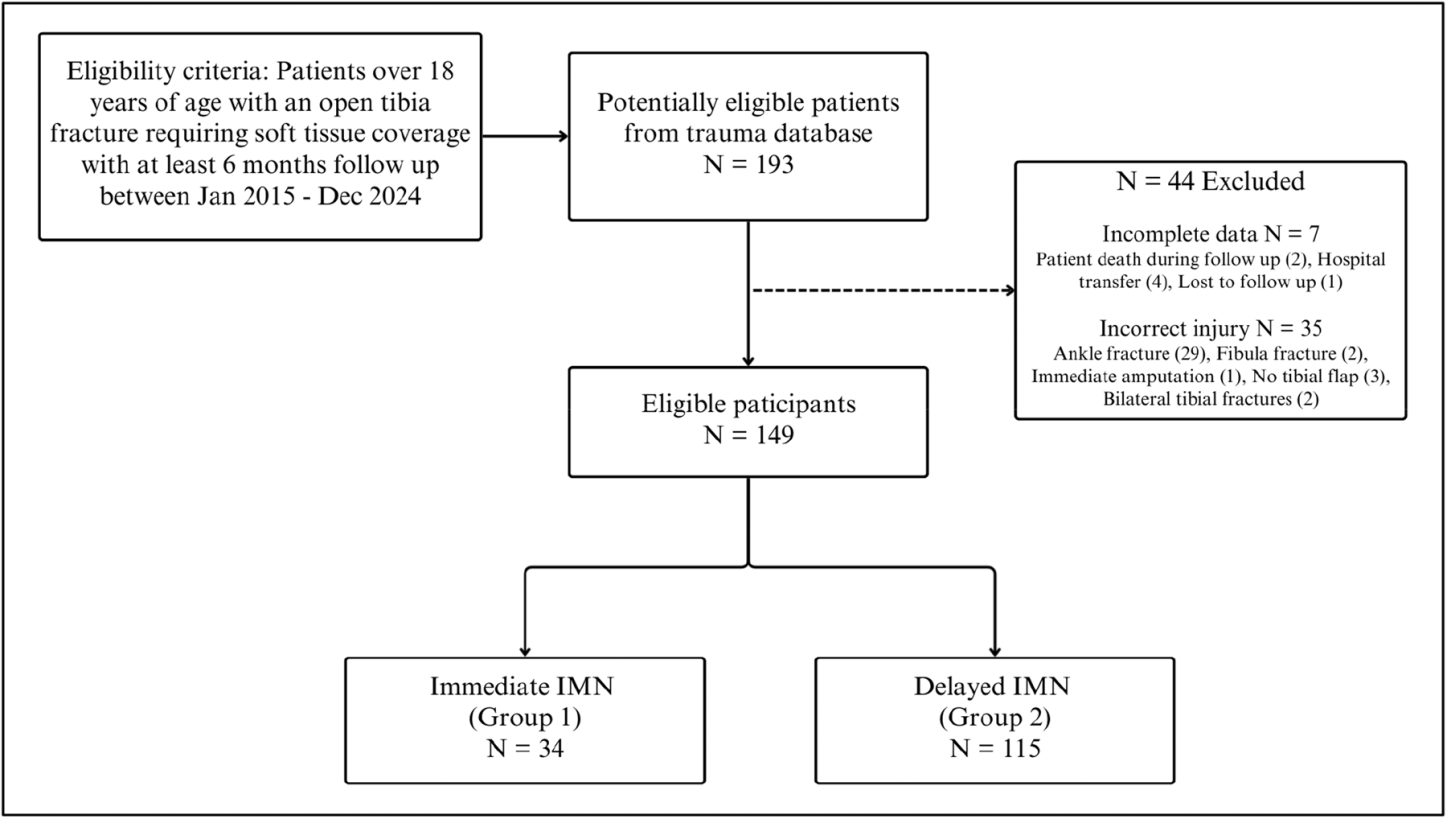
Flow diagram of participant selection.

A total of 193 patients were drawn from the trauma database who had a grade IIIB or IIIC open tibia fracture from 2015 to 2024 (Figure 6). A subset of 149 of these patients were appropriate for this study. Of these, 34 (23%) patients had an immediate IMN (Group 1) and 115 (77%) had a delayed IMN (Group 2). The patients' age, gender, smoking status and Injury Severity Score (ISS) were not significantly different between the two groups (Table 1). Group 1 contained two GA IIIC injuries (5.9%), while Group 2 had 12 GA IIIC injuries (10%). Days to flap coverage did not show a significant difference between the two groups (*p* = 0.179); however, median length of hospital stay did (*p* = 0.001). The two groups had a similar distribution of mechanism of injury, but a statistically significant difference was observed in the AO classification between the two groups (*p* = 0.044). Group 1 had relatively fewer AO 43 C fractures compared with Group 2 (2.9%–23%); however, Group 1 had relatively more AO 42 A fractures (50%–23%). Free flaps were the most common (87%) across both groups. Gracilis and anterolateral thigh flaps accounted for the majority of free flaps, representing 47% and 28%, respectively. Free flaps were more frequently performed following delayed nailing (92%) compared with immediate nailing (71%). Gastrocnemius flaps made up the largest proportion (63%) of local flaps.

**Table 1:** Patient demographics.

Comparison	Immediate IMN–Group 1 (*N* = 34)	Delayed IMN–Group 2 (N = 115)	*p* Value
Age (years) (mean)	38 (SD ± 16)	44 (SD ± 16)	0.087^a^
Gender (males)	94%	90%	0.733^b^
Smoker	24%	39%	0.095^c^
ISS score (median)	10 (SD ± 5)	10 (SD ± 12)	0.864^a^
Length of stay (LOS) days (median)	17 (IQR 18)	28 (IQR 24)	**<0.001** a
Days to coverage (median)	7 (IQR 9)	9.0 (IQR 7)	0.179^a^
Mechanism of injury			0.271^c^
MVA	15%	18%	
MBA	41%	46%	
Falls	21%	25%	
Others	24%	10%	
AO Classification			**0.044** b
41 A	5.9%	3.5%	
41B	0	0.9%	
41 C	2.9%	5.2%	
42 A	50%	23%	
42B	12%	13%	
42 C	18%	18%	
43 A	5.9%	10%	
43B	2.9%	3.5%	
43 C	2.9%	23%	
Flap type			**0.002** b
Free flap	71%	92%	
Local	29%	8%	
Year			0.318^c^
2015	15%	6.1%	
2016	8.8%	10%	
2017	8.8%	12%	
2018	18%	13%	
2019	2.9%	11%	
2020	2.9%	10%	
2021	24%	10%	
2022	12%	11%	
2023	2.9%	8.7%	
2024	5.9%	7.0%	

*Note*: Bold values indicate statistically significant at *p* < 0.05.

Abbreviations: IM, intramedullary nail; MVA, motor vehicle accident; IQR, interquartile range; MBA, motor bike accident; SD, standard deviation.

a Mann–Whitney *U* test.

b Fisher′s exact test.

c *χ* ^2^ test.

### Outcomes

**Figure 7:**
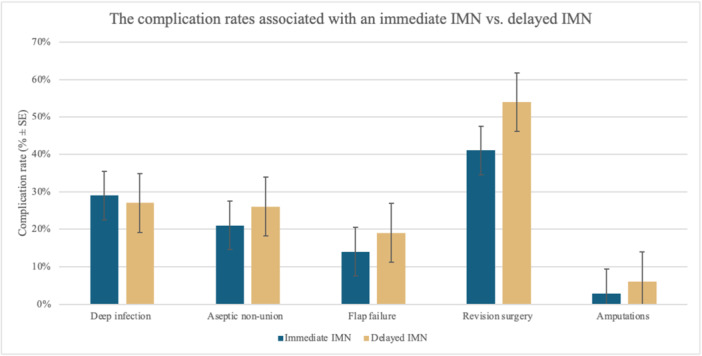
The complication rates associated with an immediate intramedullary nail (IMN) versus a delayed IMN.

The *χ* ^2^ analysis result is demonstrated in Figure 7 and Table 2. The rate of deep infection, aseptic nonunion and revision surgery showed no significant difference between patients in Groups 1 and 2. Of the 10 patients with deep infection in the immediate nailing group, six (60%) were AO class 42 A with the remainder being AO 41 A, 42B, 43B, and 43 C. In the delayed nailing group, six of the 31 patients with deep infection had an AO 42 A injury (20%), six had a 42 C (20%), and eight had a 43 C injury (26%). A majority of deep infections were multiorganism infections in the immediate nailing group with the most common organisms in the delayed nailing group being multiorganisms (34%), *Serratia marcescens* (27%) and *Staphylococcus aureus* (24%).

**Table 2:** The complication rates associated with an immediate IMN versus delayed IMN.

Comparison	Immediate IMN—Group 1 (*N* = 34)	Delayed IMN—Group 2 (*N *= 115)	*p* Value
Deep infection	29% (10)	27% (31)	0.778^a^
Aseptic nonunion	21% (7)	26% (30)	0.514^a^
Flap failure	14% (5)	19% (22)	0.556^a^
Revision surgery	41% (14)	54% (62)	0.192^a^
Amputations	2.9% (1)	6.1% (7)	0.475^a^

Abbreviation: IMN, intramedullary nail.

a *χ* ^2^ test.

Seven accounts of nonunion were recorded in Group 1, of which four (57%) had revision surgery. Of the 30 patients with nonunion in Group 2, 21 (70%) required revision surgery. Deep infection, nonunion and revision surgery were the most common complications in both groups. The single patient in Group 1 who received an amputation had chronic osteomyelitis. Of the seven that received an amputation, five had osteomyelitis and two had flap failure. A forward stepwise binary logistic regression was also performed to assess deep infection rates between groups, incorporating smoking status, AO classification (diaphyseal/proximal vs. distal) and days to flap coverage. The analysis demonstrated no significant difference in infection rates between immediate (Group 1) and delayed (Group 2) tibial nailing (odds ratio [OR]: = 1.14, 95% confidence interval [CI]: 0.46–2.85, *p* = 0.780).

A post hoc subgroup analysis using binary logistic regression was performed to examine deep infection in AO 42 and AO 43 fractures individually, as well as the length of hospital stay (LOS) between Group 1 and Group 2. Analysis of AO 41 was not performed due to the very low infection sample size (*n* = 7). No statistically significant associations were observed in any of the subgroups, controlling for age, smoking status, fracture location and flap type (AO: 42: OR = 1.12, 95% CI: 0.38–3.28, *p* = 0.843; AO: 43: OR = 1.48, 95% CI: 0.16–13.81, *p* = 0.731; LOS: OR = 0.987, 95% CI: 0.969–1.005, *p* = 0.153).

## DISCUSSION

The results of this study suggest that immediate IMN with delayed soft‐tissue coverage may not increase infection, aseptic nonunion or revision surgery rates compared with a staged fix‐and‐flap approach. No statistically significant differences were observed overall (OR = 1.14, 95% CI: 0.46–2.85, *p* = 0.780), nor in AO 42 (OR = 1.12, 95% CI: 0.38–3.28, *p* = 0.843) or AO 43 (OR = 1.48, 95% CI: 0.16–13.81, *p* = 0.731) subgroups. Immediate IMN was associated with shorter unadjusted hospital length of stay (28 vs. 17 days, *p* < 0.001); however, this effect lost significance after adjusting for age, fracture location, smoking status and flap type (OR = 0.987, 95% CI: 0.969–1.005, *p* = 0.153). Given the limited events and wide confidence intervals, these findings should be interpreted as preliminary and hypothesis‐generating rather than definitive.

Early studies examining IMN without immediate flap coverage are limited. A small series [12] featuring six tibial fractures reported no deep infections in GA IIIB fractures, while a larger cohort [8] of 161 GA III patients (AO 41–43) demonstrated acceptable outcomes with immediate nailing and flap coverage within 14 days. Interestingly, these studies suggest that the interval between definitive fixation and soft‐tissue coverage may be a stronger predictor of infection than time from initial injury, supporting coordinated ‘fix‐and‐flap’ approaches.

Patterns were observed in fracture types receiving immediate or delayed tibial nailing that aligned with clinical decision‐making. For example, the decision to delay tibial nailing was more common in distal tibial fractures (AO 43). This was likely due to its increased likelihood for soft tissue complications; thinner cortex, less adjacent soft tissue and closer proximity to the ankle [15, 18]. Likewise, distal tibial fractures were more likely to receive a free flap due to the limited local soft tissue supply and poor vascularity [17, 20]. These reasons may offer an explanation to the observed increase in median hospital LOS (28 days vs. 17 days, *p* < 0.001) in the delayed nailing group.

In practice, the early delivery of definitive fixation and soft‐tissue coverage necessitates a coordinated and timely collaboration between orthopaedic and plastic surgical teams [8]. In past studies, this joint management has been well established as a key factor in improving outcomes for patients with GA type IIIB and IIIC open tibial fractures [5, 14]. Despite this, many healthcare systems continue to face significant logistical barriers to delivering this model of orthoplastic care [12]. In emergency settings, limited availability of combined orthopaedic‐plastic teams often requires orthopaedic surgeons to manage complex injuries alone, and even when both specialties are present, collaboration can be hindered by communication gaps, fragmented workflows, and a lack of standardized protocols [14].

### Limitations

This study has several limitations. First, the sample size was modest and imbalanced between groups, limiting statistical power, particularly for less common outcomes such as aseptic nonunion and revision surgery. This imbalance restricts the strength of comparisons, bias in effect estimates and increases the risk of type II error, prompting a cautious interpretation of these preliminary results. Further subgroup analysis was also limited by a small sample size. Second, the broad 10‐year study period (2015–2024) represents another important limitation. The described methods of care for each group reflect standard practice of the institution over this period; however, surgical techniques, intramedullary nail systems, perioperative protocols and postoperative care strategies may have differed in some instances. This temporal variability may have introduced unmeasured confounding that could not be fully accounted for with retrospective analysis. Third, as a retrospective study, it is inherently subject to selection bias. The reasons for surgeons opting for immediate tibial nailing with delayed soft‐tissue coverage were not documented and may have influenced treatment allocation. Fourth, the delayed nailing group reflects real‐world practice but does not necessarily represent ideal fix‐and‐flap conditions, as many patients exceeded the 7‐day window for soft‐tissue coverage recommended by the National Institute for Health and Care Excellence (NICE) guidelines [10]. This delay could independently affect outcomes outside of the confounders captured. Fifth, several clinically relevant factors including wound contamination, bone loss, peripheral vascular disease and use of anticoagulants were not consistently recorded.

For future studies, larger cohorts with balanced fracture‐type distribution are needed to validate safety and efficacy. Additionally, prospective data would allow standardized documentation of treatment allocation rationale, perioperative protocols and further control of confounders. Research could also investigate how flexible staging affects operative scheduling, ortho‐plastic collaboration and patient throughput. Lastly, researchers should evaluate functional recovery, patient satisfaction and healthcare costs to provide a more holistic assessment of immediate versus delayed tibial nailing.

## CONCLUSION

In this retrospective cohort of GA IIIB/C tibial fractures, immediate intramedullary nailing within 24 h with delayed flap coverage was not associated with higher infection, nonunion, revision surgery or amputation rates compared with staged fix‐and‐flap. However, given limitations such as sample size constraints, these findings are preliminary only. Immediate intramedullary nailing and delayed flap coverage may be a clinical alternative to the staged fix‐and‐flap approach, but larger prospective studies are needed to confirm safety and guide clinical practice.

## AUTHOR CONTRIBUTIONS

**Emily Morris:** Concept design; acquisition of data; statistical analysis; drafting of manuscript; final approval of manuscript. **Jarrod Younger:** Acquisition of data; statistical analysis; final approval of manuscript. **Matthew Hope:** Interpretation of data; review of intellectual content; final approval of manuscript. **Richard Steer:** Interpretation of data; review of intellectual content; final approval of manuscript. **Ahmed Mahmoud:** Concept design; acquisition of data; interpretation of data; review of intellectual content; final approval of manuscript.

## CONFLICT OF INTEREST STATEMENT

The authors declare no conflicts of interest.

## ETHICS STATEMENT

All procedures performed in studies involving human participants were in accordance with the ethical standards of the institutional and/or national research committee and with the 1964 Helsinki Declaration and its later amendments or comparable ethical standards. Ethical approval for chart review and long‐term follow‐up was obtained from the Hospital Human Research Ethics Committee (Approval number LNR/2019/QMS/58750).

## Supporting information

Supplementary Information

## Data Availability

The data that support the findings of this study are available from the corresponding author upon reasonable request.
