# Supplementary material for: Intramedullary nail prior to flap coverage may not increase complications in Gustilo–Anderson Grade IIIB and IIIC open tibial fractures: A retrospective study
Source: J Exp Orthop. 2025 Nov 14;12(4):e70522. doi: 10.1002/jeo2.70522 (PMC12616495; doi:10.1002/jeo2.70522)
Supplement: Supplementary file 1 — Supplementary Information [file JEO2-12-e70522-s001.pdf]

Online Resource 1: STROBE Statement—Checklist of items that should be included in reports of ***cohort studies***

Article title: Intramedullary Nail Prior to Flap Coverage May Not Increase Complications in Gustilo-Anderson Grade IIIB and IIIC Open Tibial Fractures: A Retrospective Study

Journal name: Journal of Experimental Orthopaedics

	Item No	Recommendation	Lines	Explanation
**Title and abstract**	1	(*a*) Indicate the study's design with a commonly used term in the title or the abstract	Page 1, line 1-2	"Intramedullary Nail Prior to Flap Coverage Does Not Increase Complications in Gustilo-Anderson Grade IIIB and IIIC Open Tibial Fractures: **A Retrospective Study**"
		(*b*) Provide in the abstract an informative and balanced summary of what was done and what was found	Page 1, lines 4-26	Methods, results and conclusion briefly summarised in abstract.
**Introduction**				
Background/rationale	2	Explain the scientific background and rationale for the investigation being reported	Page 2, lines 29-50	See Introduction
Objectives	3	State specific objectives, including any prespecified hypotheses	Page 2, lines 51-54	"This study aims to challenge the existing practice of the simultaneous 'fix-and-flap' approach to determine whether immediate nailing with delayed flap coverage can serve as an acceptable alternative for the management of severe open tibial fractures"
**Methods**				
Study design	4	Present key elements of study design early in the paper	Page 3, line 57	See Methods section
Setting	5	Describe the setting, locations, and relevant dates, including periods of recruitment, exposure, follow-up, and data collection	Page 3, lines 57-64	See Methods section
Participants	6	(*a*) Give the eligibility criteria, and the sources and methods of selection of participants. Describe methods of follow-up	Page 3, lines 65-78	See Methods section
		(*b*) For matched studies, give matching criteria and number of exposed and unexposed	n/a	

Variables	7	Clearly define all outcomes, exposures, predictors, potential confounders, and effect modifiers. Give diagnostic criteria, if applicable	Page 4, lines 79-84<br>Page 7, lines 114-124	See Methods section
Data sources/ measurement	8*	For each variable of interest, give sources of data and details of methods of assessment (measurement). Describe comparability of assessment methods if there is more than one group	Page 4, line 85-91<br>Page 5, line 93-100<br>Page 6, line 104-112	Patient records, imaging notes and surgical notes
Bias	9	Describe any efforts to address potential sources of bias	Page 7, line 114-124	Control of confounders, clear inclusion/exclusion criteria and inclusion of all eligible records within time-period.
Study size	10	Explain how the study size was arrived at	n/a	As this was a retrospective study, the study size was determined by the number of eligible patients available in the database during the study period; no formal sample size calculation was performed.
Quantitative variables	11	Explain how quantitative variables were handled in the analyses. If applicable, describe which groupings were chosen and why	Table 1	See Methods and Results section.
Statistical methods	12	(*a*) Describe all statistical methods, including those used to control for confounding	Page 7, line 114-124	See Methods section
		(*b*) Describe any methods used to examine subgroups and interactions	Page 8, line 159-164	See Results section. Binary logistic regression was used to control for any confounders.
		(*c*) Explain how missing data were addressed	Fig. 6	Excluded if data missing
		(*d*) If applicable, explain how loss to follow-up was addressed	Fig. 6	Excluded if less than 6 months follow-up.
		(*e*) Describe any sensitivity analyses	Page 7, line 114-124	See Methods section
**Results**				
Participants	13*	(a) Report numbers of individuals at each stage of study—eg numbers potentially eligible, examined for eligibility, confirmed eligible, included in the study, completing follow-up, and analysed	Fig. 6	See Results section
		(b) Give reasons for non-participation at each stage	Fig. 6	See Results section
		(c) Consider use of a flow diagram	Fig. 6	See Results section

Descriptive data	14*	(a) Give characteristics of study participants (eg demographic, clinical, social) and information on exposures and potential confounders	Table 1	See Results section
		(b) Indicate number of participants with missing data for each variable of interest	Fig. 6	See Results section
		(c) Summarise follow-up time (eg, average and total amount)	Page 4, line 80-81	See Methods section
Outcome data	15*	Report numbers of outcome events or summary measures over time		n/a
Main results	16	(*a*) Give unadjusted estimates and, if applicable, confounder-adjusted estimates and their precision (eg, 95% confidence interval). Make clear which confounders were adjusted for and why they were included	Table 1<br>Table 2<br>Page 8, line 144-164	See Results section
		(*b*) Report category boundaries when continuous variables were categorized		n/a
		(*c*) If relevant, consider translating estimates of relative risk into absolute risk for a meaningful time period		n/a
Other analyses	17	Report other analyses done—eg analyses of subgroups and interactions, and sensitivity analyses	Page 8, line 159-164	See Results section
**Discussion**				
Key results	18	Summarise key results with reference to study objectives	Page 9, line 167-174	See Discussion section
Limitations	19	Discuss limitations of the study, taking into account sources of potential bias or imprecision. Discuss both direction and magnitude of any potential bias	Page 10, line 196-211	See Discussion section
Interpretation	20	Give a cautious overall interpretation of results considering objectives, limitations, multiplicity of analyses, results from similar studies, and other relevant evidence	Page 10, line 196-211	See Discussion section
Generalisability	21	Discuss the generalisability (external validity) of the study results	Page 10, line 196-211	See Discussed section.
**Other information**				
Funding	22	Give the source of funding and the role of the funders for the		Nil funding sources

	present study and, if applicable, for the original study on which the present article is based

*Give information separately for exposed and unexposed groups.

Note: An Explanation and Elaboration article discusses each checklist item and gives methodological background and published examples of transparent reporting. The STROBE checklist is best used in conjunction with this article (freely available on the Web sites of PLoS Medicine at http://www.plosmedicine.org/, Annals of Internal Medicine at http://www.annals.org/, and Epidemiology at http://www.epidem.com/). Information on the STROBE Initiative is available at http://www.strobe-statement.org.
